# Connecting the bibliographic-directed citation networks of translational research and implementation science

**DOI:** 10.1017/cts.2025.11

**Published:** 2025-02-27

**Authors:** Rose Hennessy Garza, Jane E. Mahoney, Morgan Burns, Andrew Quanbeck

**Affiliations:** 1 Joseph J Zilber College of Public Health, University of Wisconsin-Milwaukee, Milwaukee, WI, USA; 2 Department of Family Medicine and Community Health, School of Medicine and Public Health, University of Wisconsin-Madison, Madison, WI, USA

**Keywords:** Implementation science, translational science, CTSA, bibliometrics, citation network analysis, VosViewer

## Abstract

**Introduction:**

Translational science and implementation science are two disciplines that integrate scientific findings into practice within healthcare. One method to assess the integration of these fields is to review the academic crossover between the disciplines with respect to shared citations in the peer-reviewed literature.

**Methods:**

This paper used direct citation network analysis to identify potential conceptual gaps and connections between the literature in implementation science and translational science. Bibliographic references were downloaded from Web of Science to create directed citation network maps in VosViewer. Heat maps visualized the top cited literature in each field.

**Results:**

A literature search yielded 6,111 publications in translational science and 7,003 publications in implementation science. When all publications were combined in a directed citation network map, two separate groups of publications emerged, representing the two fields of implementation science and translational science. When the top 50 cited translational science publications were combined with implementation science publications, 14% had a 100%+ increase in citation links, 44% had a mean increase of 2.4%, and 42% shared no links. When the top 50 cited implementation science publications were combined with translational science publications, 2% had a 100%+ increase in citation links, 92% had a 3.3% mean increase, and 6% had no shared links.

**Conclusions:**

Results suggest moderate academic overlap in the way published authors cite each other between translational science and implementation science. We hope the implications of this paper may promote continued collaborations between these fields to disseminate lessons learned and bridge research into practice more efficiently.

## Introduction

Translational science and implementation science are two disciplines that focus on moving scientific knowledge from discovery into practice within healthcare organizations and public health. Translation is defined by the National Center for Advancing Translational Science (NCATS) as “the process of turning observations in the laboratory, clinic, and community into interventions that improve the health of individuals & the public [1],” and translational science is “the field of investigation which seeks to understand the scientific and operational principles underlying each step of the translational process [1].” Implementation is defined by the National Institutes of Health as, “the adoption and integration of evidence-based health interventions into clinical and community settings for the purposes of improving delivery, outcomes, and individual & population health [2],” and implementation science is “the scientific study of methods to promote the systematic uptake of research findings and other evidence-based practices into routine practice, and, hence, to improve the quality and effectiveness of health services [3].”

Together, translational and implementation sciences aim to bridge the gap between what *works* to address health and what is *done* to address health. Scientific inquiry using these overlapping disciplines remains essential to address the gap between the creation of clinical knowledge and its integration into healthcare. Bridging translational science and implementation science may facilitate improvement in health care delivery. These fields connect within NCATS’ translational science spectrum, a nonlinear model depicting the progression of research from basic sciences to interventions that improve health [4]. Key stages of the spectrum include clinical implementation of health interventions and public health. These stages link implementation science directly to translational science, with implementation research considered one stage of translational research while also informing additional stages.

**Figure 1.**
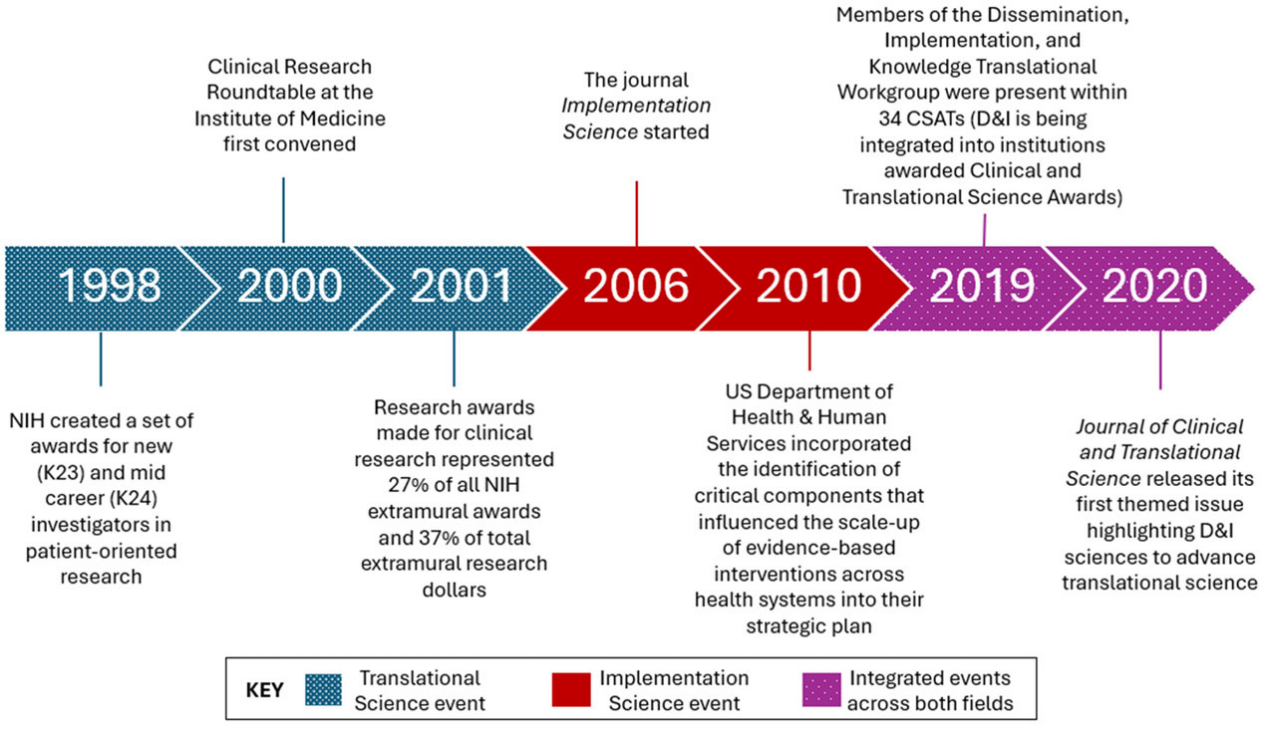
Timeline of key events in implementation science and translational science. (a) Translational science directed citation network. (b) b) implementation science directed citation network.

A progression of efforts to advance implementation science and translational science has occurred over the past 20 years. Since 2001, numerous financial contributions and grants have been made available for clinical implementation research [5], increasing knowledge production and helping to facilitate the launch of the journal *Implementation Science* in 2006 [3]. Beginning in 2010, components that influence the scale-up of evidence-based interventions across health systems were incorporated into the US Department of Health and Human Services strategic plan [6]. Currently, implementation science has been formally integrated in over half of the Clinical and Translational Science Award recipients (CTSA hubs), with 34 hubs represented on the *Dissemination, Implementation, and Knowledge Translation Working Group* as of 2019 [7] and continued recommendation by scholars to continue this trend [8]. The national working group *Advancing Dissemination and Implementation Sciences within CTSAs* advances translational research in CTSA hubs through capacity building to incorporate principles and strategies of implementation science [9]. In July 2020, the *Journal of Clinical and Translational Science* released its first themed issue highlighting implementation science to advance translational research [10]. Reflecting federal priorities, in July 2021, the funding award PAR-21-293 required all CTSA hubs to actively engage in implementation activities [11]. These and additional efforts are highlighted in Fig. 1.

Our study examines the literature that connects translational science and implementation science to assist leaders at medical research centers throughout the U.S. implement their research findings into clinical practice. This work builds upon recommendations by McKibbon et al [12], who highlighted the need for easier access to literature across disciplines for knowledge translation to progress. The aim of this paper was to examine the bibliographic bridge between the sciences of translation and implementation by 1) determining key interconnected literature in each field and 2) documenting bridging publications between fields while quantifying the overlap of scientific inquiry within translational science and implementation science. Our overarching goal was to increase our understanding of how these two fields are connected in the literature by presenting a more visual, numeric, and tangible understanding of overlap and separation between the research fields and authors. Recognizing bibliographic overlap can provide resources to scholars in both fields to help cultivate a more cohesive academic community working in less isolation and more unity to streamline and share lessons learned in knowledge translation.

## Materials and methods

We used directed citation network analysis to create directed citation network maps of publications in translational science, publications in implementation science, and a combination of these publications to examine overlap in the literature of the two fields. While the overall aim of our paper differs in scope, our methodology parallels the work of Fort et al [13] to connect highly intercited literature within translational research. We expanded upon their work to examine connections between translational science and implementation science literature. Our methods included four main components: (1) a systematic search to obtain literature in translational science and implementation science; (2) directed citation network analysis to create directed citation network maps that connect publications through shared citation links; (3) construction of heat maps to visually display global citation data for the most cited publications within translational science and implementation science; and (4) a quantitative comparison to assess interconnections between translational science and implementation science via citation links.

### Systematic literature search

To obtain publications in translational science and implementation science, we used identical search terms by Fort et al. [13] for translational science and adapted the search terms by Davis & D’Lima [14] for implementation science. Using database filters, articles, review articles, books, or book chapters published within 2022 or any year beforehand were identified in *Web of Science* by searching for key terms in the title, author-selected keywords, or Keywords Plus, which are words or phrases that frequently appear in the titles of an article’s references [15]. The translational science search yielded 6,111 publications of articles, reviews, books, and book chapters using identical search terms from Fort et al [13]: “translational science*” OR “clinical and translational science*” OR “CTSA*” OR “translational research” OR “translation research” OR “clinical and translational research” OR “translational medicine.” For each search term, we used the same proximity operator by Fort et al. [13] to find records where the terms were within five words of each other (i.e., “translational NEAR/5 medicine” OR “translational NEAR/5 research). The proximity operator was added to yield the most comprehensive search of the field within the literature (i.e., to capture an article entitled “The science of implementation”). All publications were included and no additional screening was employed.

The implementation science search yielded 7,003 articles, review articles, books, and book chapters using adapted implementation science search terms from a systematic review by Davis & D’Lima [14]: “implementation research” OR “implementation science*” OR “improvement research” OR “improvement science” OR “dissemination science” OR “knowledge mobilisation” OR “knowledge translation.” We expanded the search by adding the NEAR/5 proximity operator to mirror the translational science search (i.e., “implementation NEAR/5 science”). Two terms from Davis & D’Lima’s [14] search were omitted: “knowledge transfer” was excluded as our focus was on health science and related literature, and the addition of this term yielded literature in business, management, computer science, and information technology. “Quality improvement” was excluded because it led to a vast literature with publications beyond the scope of implementation, including specific medical recommendations and guidelines that lacked an implementation focus. “Knowledge translation” was included as it was described by Davis & D’Lima [14] as a related and synonymous term for implementation science.

In this study, we were interested in the *science* of translation and the *science* of implementation, as compared to translational research or implementation research. Since the seminal paper by Austin [1], the NCATS has made a clear distinction between translational research and translational science – with research defined as “the endeavor to traverse a particular step of the translation process for a particular target or disease,” while science “seeks to understand the scientific and operational principles underlying each step of the translational process” [1] Conclusively, translational research studies one disease/target while translational science focuses on applications in translation for any disease, such as common causes of inefficiency or failure in translational research projects [1]. Similarly, implementation science is the overall study of methods to promote the systematic uptake of research and evidence-based practices into practice [3], while implementation research seeks to understand processes and factors associated with the successful integration of specific research and evidence-based practices within a particular setting [16]. While the distinction in terminology has improved in recent years, historically many authors used the terms “science” and “research” interchangeably. Therefore, we included both search terms (science and research) in our literature search.

### Directed citation network analysis

Citation data were obtained from the *Web of Science* title of each paper. The program *VosViewer* [17] was used to identify citation networks of publications that cite each other. A citation network is a structure of linked academic publications that uses citations from one document to connect to another. Per recommendations by Van Eck & Waltman [18], we used *VosViewer’s* default association strength normalization method within all constructed networks to normalize the strengths of the links between our publications [17]. Our directed citation network maps showed *citation links* between publications, with a connecting line or link occurring when one publication cited another in the group [17]. Each publication was represented by a circular node, with node size representing the frequency of citation links between one publication and another. *VosViewer* mathematically assigns each publication to a cluster with a different color. Clusters are sets of documents included in the directed citation network map that are substantially more connected to each other compared to others in the group, with each publication belonging to only one cluster at most.

**Figure 2.**
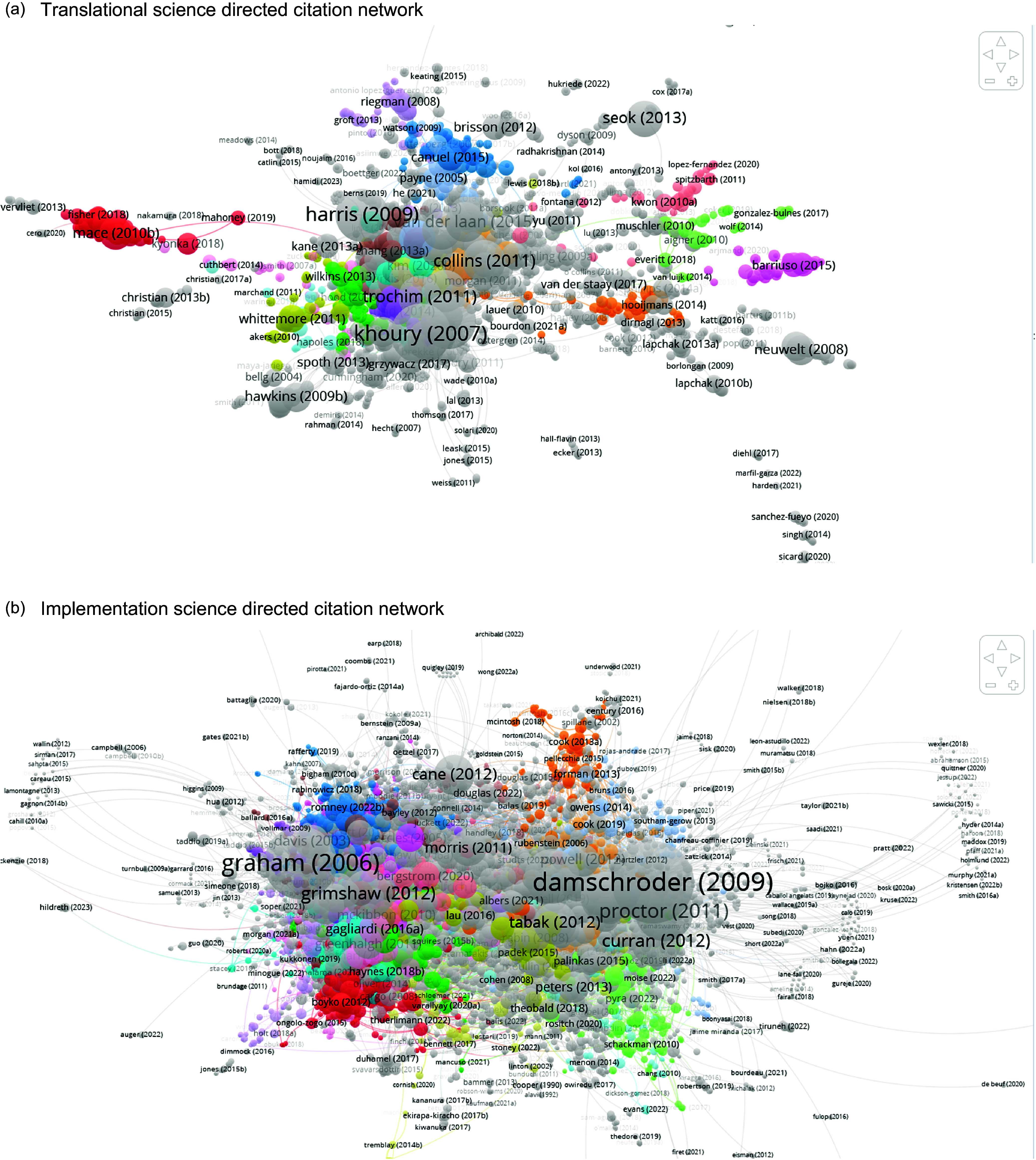
Separate citation networks with citation links between publications. *Colors indicate clusters determined by VosViewer. Node size is based on the number of citation links with other publications in the network (ie- bigger nodes have more citation links with the other publications in the network).

**Figure 3.**
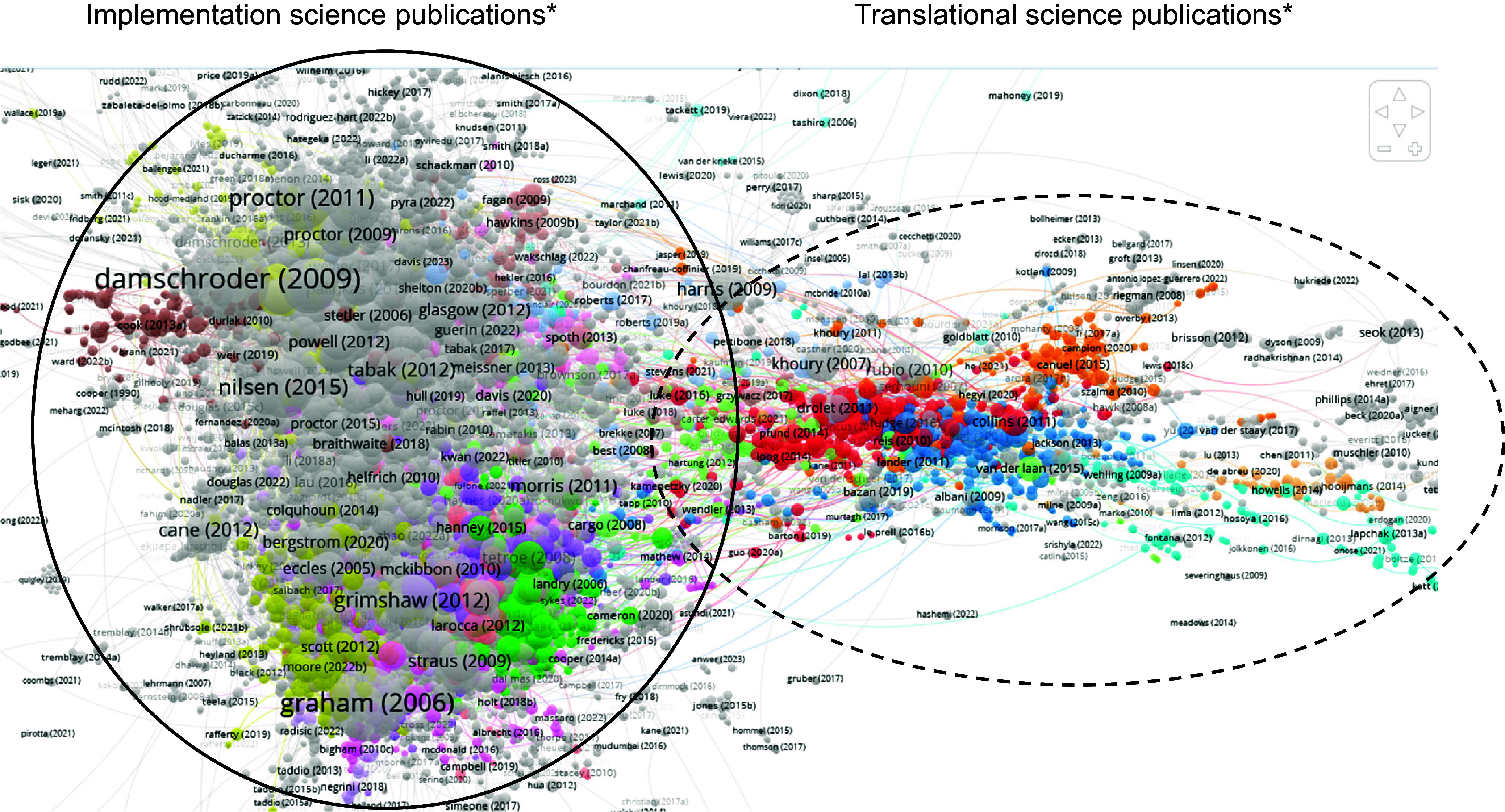
Combined implementation science and translational science citation network. *The majority of publications in the enclosed circles come from the separate implementation science (solid) and translational science (dashed) network analyses. colors indicate clusters determined by VosViewer. Node size is based on the number of citation links with other publications in the network (ie- bigger nodes have more citation links with the other publications in the network).

Three total directed citation network maps were constructed. The first and second directed citation network maps examined publications in translational science (Fig. 2a) and implementation science (Fig. 2b), respectively. All publications obtained in the literature search for each respective topic were used to create each directed citation network map. To compare the citation linkages between publications in translational science and implementation science, a third directed citation network map was created, which combined all publications from the literature searches in translational science and implementation science (Fig. 3).

### Heat maps of global citations

Two heat maps were constructed to visually indicate how frequently the 50 most cited papers in each field were annually cited. One heat map was created for implementation science and the other for translational science. Citations were tabulated from Web of Science and were presented annually and in cumulative totals through the end of 2022. Global citations arose from anywhere in the literature and are not limited to the directed citation network maps. Heat maps use color to represent higher values of data with darker colors and lower values with a lighter color, making it easier to detect patterns in the data [19,20]. The light-colored yellow boxes indicated less citations, while dark-colored red boxes indicated more citations.

### Percent change in citation links

To provide an understanding of how the most cited literature overall is cited in either translational science, implementation science, or among both fields, additional data were included in the heat maps. The number of citation links between each of the top 50 publications was provided to show the field-respective links in each directed citation network map and the number of links in the *combined* directed citation network map. The change in links was then calculated to demonstrate the percent increase in citation links. For example, a hypothetical publication in translational science might be cited 1,000 times in the overall literature. There may be 400 citation links between this article and others in the translational science directed citation network map. When examining the article in the combined directed citation network map, there may be 450 citation links, demonstrating an additional 50 citation links between translational science and implementation science for this publication. This would translate to a 12.5% increase in this fictitious example.

## Results

### Separate citation networks

Fig. 2a depicts the directed citation network map for translational science, and Fig. 2b depicts the directed citation network map for implementation science. The citation network of 6,111 possible publications in the search pool for translational science had a largest component that included 2,360 publications. Only these 2,360 were included in the analysis, and the other 3,751 publications were not assigned to a cluster, following the analysis and reporting provided by Van Eck & Waltman, the developers of the VosViewer program used for the analysis and visualization [21]. (A publication is only included in the map if it is cited by another publication or cites another publication within the network.) There were 4,894 links, or shared citations, between the included publications. The largest nodes were publications that have the most citation links to others in the network and included Khory et al. [22] (n = 118 links), followed by Rubio et al. [23] (n = 101 links) and Harris et al. [24] (n = 92 links) (see Supplementary Material Appendix A1 for the 50 most linked publications in this directed citation network along with total citations for each publication). Khory et al. [22] presented a four-phrase framework for the continuum of translational research in genomics (i.e., phase 1 through phase 4). Rubio et al. [23] considered the needs for a workforce in translational research, posited objectives for trainees, and provided considerations for evaluation of training programs. Harris et al. [24] were the seminal article describing the research electronic data capture program (REDCap) used to collect, store, and analyze data.

In Fig. 2b, the citation network of 7,003 possible publications in the search pool for implementation science had a largest component that included 5,754 publications. Only these 5,754 were included in the analysis, and the other 1,249 publications were not assigned to a cluster. There were 25,367 links between the included publications. The largest nodes in the network were publications by Damschroder et al. [25] (n = 1,277 links), followed by Graham et al. [26] (n = 969 links) and Proctor et al. [27] (n = 561 links) (see Supplementary Material Appendix A2 for the 50 most linked publications in this directed citation network along with total citations for each publication). Damschroder et al. [25] presented the development of the Consolidated Framework for Implementation Research (CFIR). Graham et al. [26] provided definitions for terminology related to moving knowledge into action and presented a conceptual framework to integrate knowledge creation with application. Proctor et al. [27] presented eight implementation outcomes to assist in the evaluation of implementation differently from service system or clinical treatment outcomes.

### Combined Analysis: Comparing translational science and implementation science

As an aim of this study was to explore citation linkages between implementation science and translational science, all publications from the translational science literature search and implementation science literature search were combined to create a directed citation network map. In Fig. 3, the citation network of 12,988 possible publications had a largest component that included 8,247 publications. Only these 8,247 were included in the analysis, and the other 4,741 publications were not assigned to a cluster. Previously, there were 4,894 links in the translational science directed citation network map and 25,367 links in the implementation science network. The combined network yielded 31,400 citation links among the 8,247 publications that cited or were cited by at least one other publication in the combined pool, representing a 3.76% increase in linkages when combining the literature from the two separate fields. The visual representation within the combined directed citation network map depicts the publications in two larger groups that mirror the separate directed citation network maps for translational science from Fig. 2a (solid-line circle in Fig. 3) and implementation science from Fig. 2b (dotted-line circle in Fig. 3). While publications were more likely to be situated closer to those in their originating directed citation network, the visualization shows publications in the implementation science group that linked with publications in the translational science group, and vice versa.

### Citation frequency

Two heat maps were created. The first included the top 50 most cited publications from the translational science literature search. The second included the top 50 most cited publications from the literature search for implementation science.

**Figure 4.**
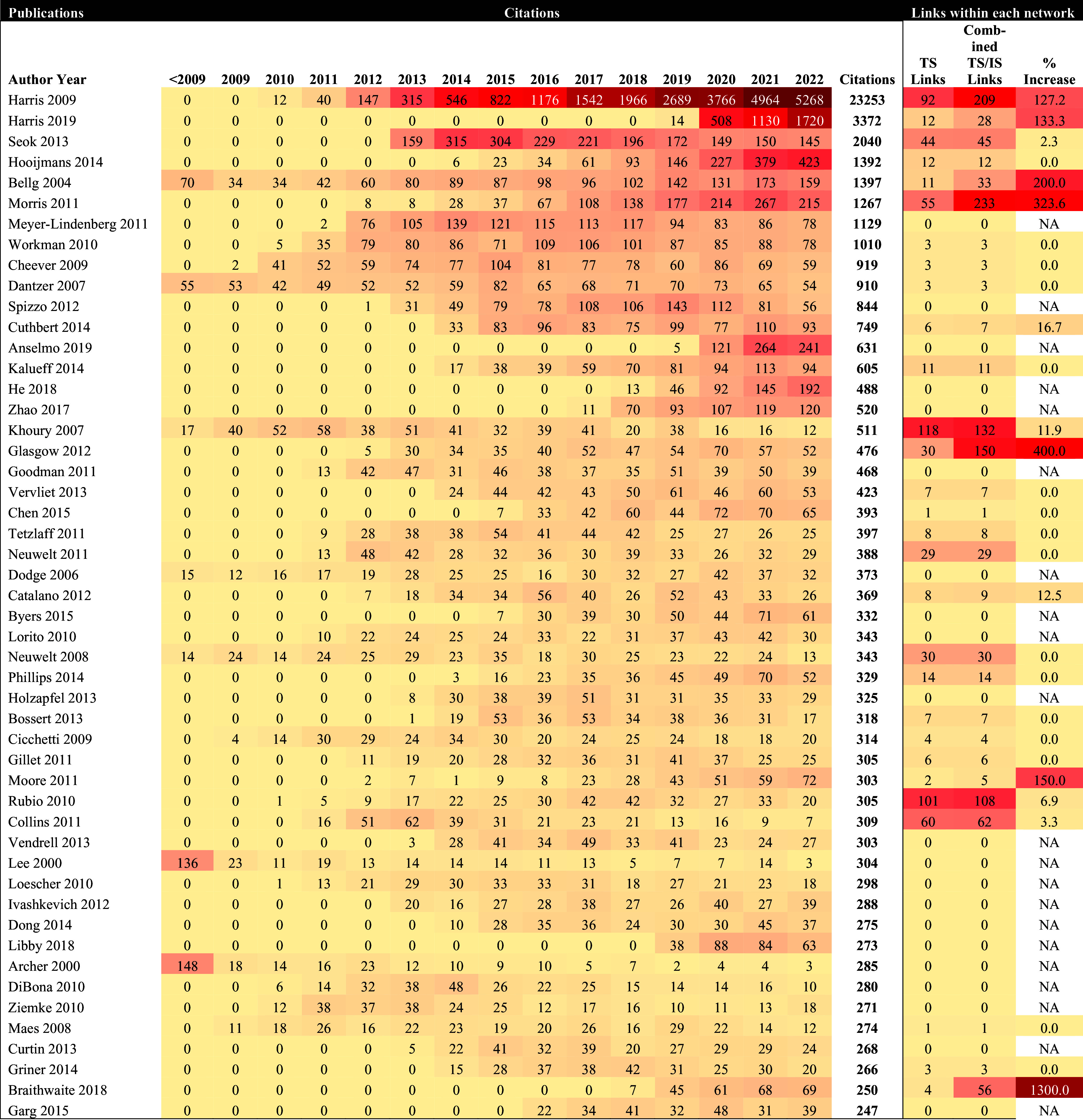
Annual overall citations for top-50 most cited publications in the translational science network.

Fig. 4 depicts the heat map of the top 50 overall most-cited publications from the translational science literature pool (see Supplementary Material Appendix A1 for the 50 most linked publications in the translational science directed citation network of Fig. 2A along with total citations for each publication). In total, the 6,111 translational science publications were cited 182,747 times. The top 50 most cited publications were cited 51,173 times, with a median of 356 citations per publication (min = 247, max = 23,253). The top most cited publications were Harris et al. [24] (n = 23,253 citations) and Harris et al. [28] (3,372), two publications that describe the research electronic data capture (REDCap) and the REDCap consortium. Seok et al. [29] (n = 2,040 citations), the third most cited publication, demonstrated a poor correlation in genomic responses between mouse models and human inflammatory diseases. Five additional publications were cited more than 1,000 times, including Hoojimans et al. [30], Bellg et al. [31], Morris et al. [32], Meyer-Lindenberg et al. [33], and Workman et al. [34].

**Figure 5.**
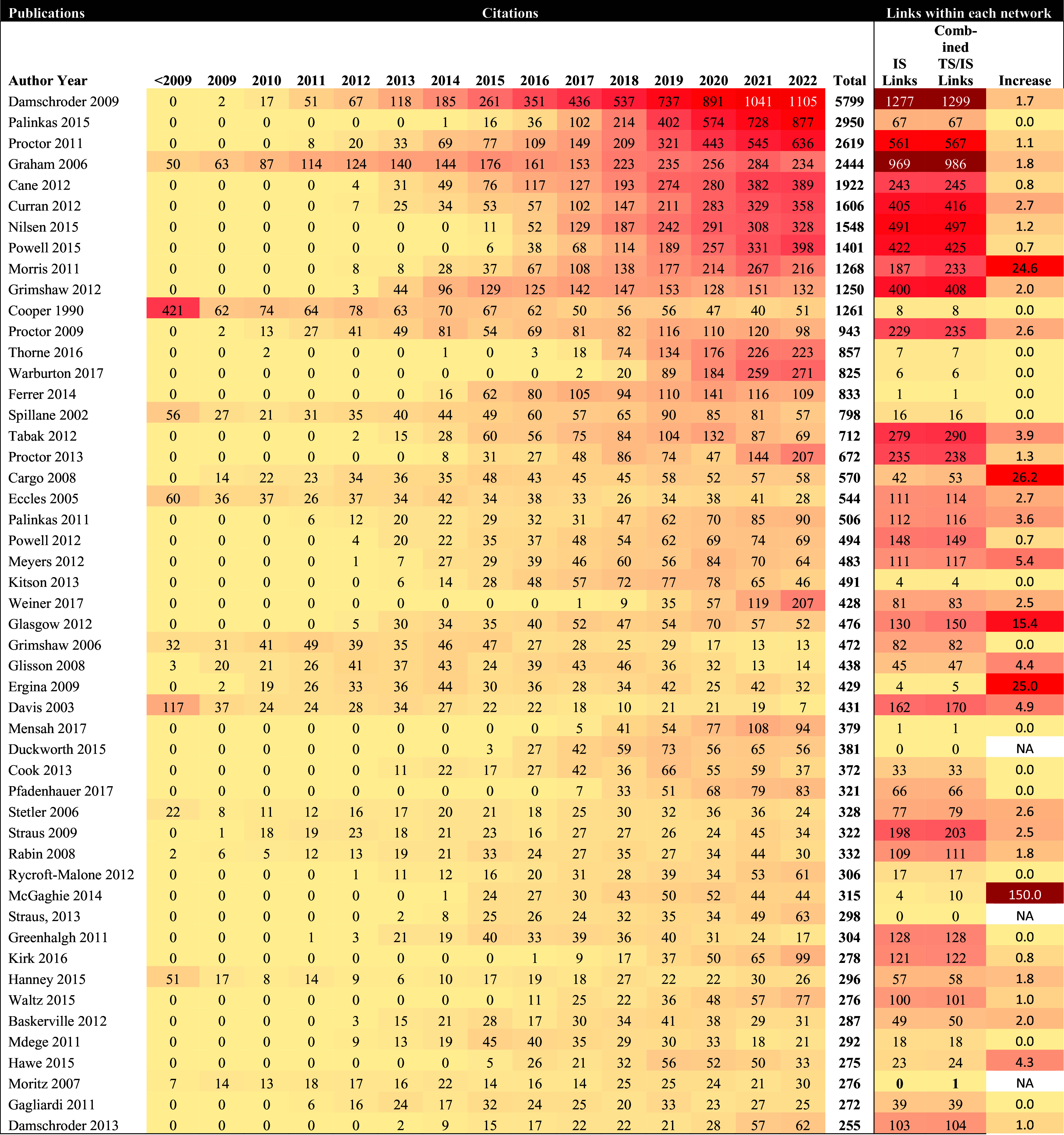
Annual overall citations for the top 50 most-cited publications in the implementation science network.

Fig. 5 depicts the heat map of the top 50 most-cited publications from the implementation science literature search pool (see Supplementary Material Appendix A2 for the 50 most linked publications in the translational science directed citation network of Fig. 2B along with total citations for each publication). In total, the 7,003 publications were cited 156,363 times. Overall, the 50 most-cited publications were cited 41,635 times, with a median of 474 citations per paper (min = 255, max = 5,799). Damschroder et al [25] were the most cited paper within the group (n = 5,799 citations), followed by Palinkas et al. [35] (n = 2,950) and Proctor et al. [27] (n = 2,619). Palinkas et al. [35] reviewed purposeful sampling strategies in implementation research and provided recommendations for mixed methods research. Eight additional publications were cited more than 1,000 times, including Graham et al. [26], Cane et al. [36], Curran et al. [37], Nilsen et al. [38], Powell et al. [39], Morris et al. [32], Grimshaw et al. [40], and Cooper et al. [41].

### Interconnections between translational science and implementation science via citation links

To quantify the overlap in citation links of publications between fields, we included three data points each for the top 50 most-cited publications in both translational science and implementation science. (1) At the end of the Fig. 4 heat map (of the top 50 most cited translational science publications), the number of citation links in the translational network from Fig. 2a was included. (2) The number of citation links for each publication in the combined network in Fig. 3 was listed next. (3) The percentage change was calculated between the citation links in the translational science directed citation network map and the combined directed citation network map. For example, the first article in the heat map of Fig. 4 is Harris et al. [24]. This article was cited 23,253 times anywhere in the global literature: (1) It shared 92 citation links with other publications in the translational science directed citation network map and (2) it shared 209 citation links with publications in the combined directed citation network map. (3) The additional links to implementation science publications represented an increase of 127.2%. The same process and calculation of percent change was repeated for the implementation science heat map in Fig. 5.

In the translational science heat map (Fig. 4), 21 of the 50 publications did not have any citation links in either directed citation network map. This signifies that while these 21 publications are cited highly in the overall literature, they are not cited by or citing other publications included in the translational science or implementation science search pool (based on our search criteria). This suggests that 42% of the top cited publications in translational science are being cited by disciplines other than translational science or implementation science. Seven of the top 50 publications from the translational science search pool had over a 100% increase in citation links when combined with publications from the implementation science search pool: Braithwaite et al. (1300% increase) [42], Glasgow et al. [43] (400% increase), Morris et al. [32] (324% increase), Bellg et al. [31] (200% increase), Moore et al. [44] (150% increase), Harris et al. [28] (133% increase), and Harris et al. [24] (127% increase). The remaining publications in the translational science heat map (n = 22) had a 2.4% average percentage increase in citation links when combined with the implementation science publications in the combined directed citation network map (mode = 0%, median = 0%, max = 16.7%).

From the implementation science heat map (Fig. 5), only two publications were not included in the implementation science directed citation network map. One of these publications, Moritz & Woodward [45] lacked citation links with other implementation science publications, but gained one citation link when combined with the translational science literature. McGaghie et al. [46] were the only publication with a 100%+ increase in citation links between the implementation science directed citation network map and the combined directed citation network map (from *n* = 4 citation links to *n* = 10 citation links). The remaining publications in the implementation science heat map (n = 47) had an average percentage increase of 3.3% in citation links when combined with the translational science literature (mode = 0%, median = 1.2%, max = 26.2%).

## Discussion

This paper examined the most highly cited publications within and across implementation science and translational science to assess shared links, identify top cited publications, and understand how the literature is connected. While previous studies have used citation network analysis to assess other aspects of these fields [47,48], this study was novel in using directed citation network analyses to identify the extent to which publications in implementation science and publications in translational science were linked through citations. Our study provides a contribution to the scant literature assessing the relationship between translational and implementation sciences. Leppin et al. [49] proposed an integrated framework to characterize how implementation science is situated within translational research, while Mehta et al. [8] explored the relatedness of the fields from a practical standpoint, but neither publication provided a quantifiable analysis to examine the degree of relatedness as seen in citations of the literature between the two fields.

Overall, when publications from the translational science and implementation science literature were merged within a combined directed citation network map, we found shared citation links between fields, but were still able to identify two groupings of publications, one derived from translational science keywords and one from implementation science keywords (Fig. 3). Our results demonstrated mostly small percentage increases in citation linkages among (1) commonly citated articles and (2) when combining the individual literature in translational and implementation science to create a combined directed citation network map (<4%). However, there were cases of commonly cited publications with greater increases in the percentage of linkages across fields, some with percent increases in citation links over 100% ([24,28,31,32,42–44,46].

We interpret these findings to suggest there is modest bibliographic overlap between the fields based on authors citing each other.

There are two clear examples of publications that are highly cited in the combined directed citation network map that arise from the literature in both translational science and implementation science. Harris et al. 2009 [24] is a centralized node in the combined directed citation network map that discusses the REDCap tool used in implementation and translational science research. REDCap is an example of how a tool, methodology, or framework that is highly relevant for translational research is also highly relevant for implementation research.

A second publication that is well cited in translational science and implementation science is Morris et al. [32]. This is a top cited publication in both fields. The title, “The answer is 17 years, but what is the question: understanding time lags in translational research,” directly pertains to translational research but references the 17-year gap, which clearly emphasizes implementation science. In contrast to the REDCap article, which addresses a widely recognized tool used across numerous scientific disciplines, we find that Morris et al. [32] conceptually links fields and highlights shared concerns across translational science and implementation science. While Harris et al. [23], Morris et al. [32], and six other publications [28,31,42–44,46] from among the top 50 cited publications in each separate scientific field demonstrated a large increase in citations among the combined citation network (>100% increase), many publications do not share any citation links between fields: 76% of the top 50 cited publications in translational science and 36% of the top 50 cited publications in the implementation science network reported no increases in citations when combined (this includes articles with no original linkages within their own networks)

Finally, the comparison of the separate heat maps and citation networks provided additional information on the state of these fields for consideration. Implementation science publications were more interconnected than were translational science publications based on (1) how many publications cited each other within each field and (2) how many shared links there were between fields: (1) 82.2% of publications in the implementation science directed citation network map cited each other (n = 5,754 publications from a pool of 7,003) compared to only 38.6% of publications from the translational science directed citation network map (n = 2,360 publications from a pool of 6,111). (2) Implementation science publications shared over five times as many citation links as translational science publications (n = 25,367 implementation science links; *n* = 4,894 translational science links). One possible explanation for fewer connections within the translational science citation network may be the wide range of research that falls within translational research. Less interconnectedness in translational science, compared to implementation science, may also be explained by Van der Laan et al [50], who states it is misleading to label all translational activities as one field, because “it suggests that it would suffice to perform and stimulate one type of research” to accomplish all translational aims. In contrast, implementation science in general has dealt with the later stages of research in the translational research spectrum and therefore may be expected to have a more interlinked literature.

In summary, this study adds to what is known about the literature on the inter-relatedness of citations in translational and implementation sciences [10]. We see room for growth between shared citation links in the academic literature of translational science and implementation science to align the fields together more strategically [49].

### Implications

Our study brings into question what a successful benchmark would be to represent useful integration between translational and implementation sciences based on directed citation network analysis. Unlike a methodology such as a meta-analysis that can estimate the strength of an association [51], we do not expect to see 100% increases in citations across every publication, as translational science and implementation science are not identical fields. Further, there will always be literature that is critical to advance specific research topics but is less relevant to the overall fields of translation and implementation, such as “Exosome Theranostics: Biology and Translational Medicine” [52], the 15^th^ most cited publication in our translational science literature search. However, highlighting publications that are highly cited in both search pools and identifying publications that could be applicable across fields to share tools and lessons learned could help bridge important gaps and keep the fields from developing in academic isolation.

With the July 2021 release of funding award PAR-21-293, which requires all CTSA hubs to actively engage in implementation activities, among others, this publication is timely. We believe that enhanced collaboration between translational science and implementation science can increase the efficiency of these fields to develop and translate health healthcare innovations into practice. Expanded integration of implementation science within CTSA hubs is one critical step to help accomplish this mission, in line with the recommendation by Leppin et al [49] that implementation science should be an integral sub-science of translational science [8]. Guided by Mehta et al [8], we reinforce and expand upon three recommendations made by Hwang et al [53] to advance the mission of the CTSA program using implementation science:

1. *Integrate implementation methods and designs into CTSA hubs’ existing processes that support translational research*. Our study highlights key publications of interest in implementation science that could provide opportunities for integration within translational science, including work that outlines common implementation theories, models, and frameworks [25,36,54–57]; assists in the selection and use of these in practice [38,58]; provides considerations for research design and methodology in the field [35,37,59,60] [44]; and highlights outcome and evaluation metrics [27,61–63]. Publications providing information on implementation strategies and how to select them are also available for integration into CTSA hubs [39,64–66]. This literature can assist CTSA hubs to find and utilize tools from implementation science more easily.
2. *Lead the advancement of implementation science through collaboration.* Hwang et al [53] focus on collaboration of implementation scientists with other NIH institutes. We also acknowledge the need for partnerships across disciplines, institutes, and stakeholders to maximize the usefulness of implementation science in CTSA hubs [8,53,67]. Implementation publications focused on specific clinical application areas can assist with partnerships in specific areas, for example, mental health services [68,69] or education [61,70]. Lessons learned in participatory research [71,72] may assist in collaboration across institutions and provide assistance on centering patients and end-use stakeholders in translational research. Publications across both fields also provide different perspectives to navigate shared challenges, such as complexity [42,73,74].
3. *Provide training in implementation science*. Top cited literature in implementation science provides key background knowledge and training for audiences new to implementation science. Publications by Straus et al. [75] and Rabin et al. [16] define common terminology in implementation science, providing key foundational concepts. Additional publications provide guidance for specific goals, such as improving guideline use [57] or policy implementation [76]. Training in translational research [23] can be modified to incorporate implementation science. One particular opportunity for integration may be the provision of implementation science seminars for KL2 scholars run by CTSA hubs, which currently happens at some universities.

### Limitations

Our study results are limited to publications we obtained from a Web of Science search for titles, keywords, or Keywords Plus that represent terms commonly associated within translational science and implementation science. In our initial search, we did not include the term “research” in any search terms. However, we found that many scholars have previously used “research” and “science” interchangeably and that it was necessary to include the terminology for “translational research” and “implementation research” to obtain a wide breadth of articles that also discuss science. Other publications in these fields may not include our search terms in their titles, keywords, or Keywords Plus and may have been excluded. Further, we conducted our review based on search terms from previous scholars, but misclassification of articles may still have occurred due to inconsistent use of terminology and imperfect search filters in implementation and translational sciences [16,77]. Publications that are not indexed in Web of Science would not have been obtained in our literature search, potentially excluding pertinent literature of interest. Our study was subject to a limitation that impacts all bibliographic research, i.e. citation network “lag,” in which newer publications have less time to accumulate citations. In addition to lags, a lack of normalization of citation data can lead to bias; this is particularly true for the heat maps assessing the most-cited literature overall within each field. The directed citation networks in our study used a normalization method to help correct for these issues as a preliminary start, but future research could expand this work with more advanced techniques that involve indirect citations and bibliographic coupling [78], or new metrics like *weighted direct citations* that integrate direct citations with references and co-citations [79]. Within VosViewer, we were limited to use the “largest component” for analysis within our networks, and future work would benefit from the “main component” to address the boundary specification issue in network studies. Despite these potential limitations, this study provided an initial and novel visual manner to assess citation overlap between the fields of implementation science and translational along with a quantifiable perspective of how the academic disciplines intersect in the literature.

## Conclusions

Translational science and implementation science are two disciplines that aim to integrate scientific findings into healthcare and public health. As our findings show, the disciplines overlap bibliographically across some publications, with opportunities to increase citation links across fields. Our network analysis reinforces the established notion that translational science and implementation science are conceptually integrated and that there is continued opportunity for collaboration between authors in the two fields. Future research could also assess clustering of bibliographic directed citation network maps within these fields to explore connectedness, themes over time, and further leverage opportunities for collaboration. We hope this paper provides a continued platform to support bibliographic integration of the two fields, shares resources to further this effort, and advances collaboration to subsequently improve clinical practice and the public’s health.

## Supporting information

Hennessy Garza et al. supplementary material Hennessy Garza et al. supplementary material

## Supplementary material

The supplementary material for this article can be found at https://doi.org/10.1017/cts.2025.11.
