# Supplementary material for: Connecting the bibliographic-directed citation networks of translational research and implementation science
Source: J Clin Transl Sci. 2025 Feb 27;9(1):e64. doi: 10.1017/cts.2025.11 (PMC11975788; doi:10.1017/cts.2025.11)
Supplement: Hennessy Garza et al. supplementary material [file S2059866125000111sup001.docx]

**Supplementary Material: Appendix**

Figure A1. Total citations of the 50* most interlinked publications from Figure 2A. Translational science directed citation network

| **Publication** | **Title** | **Journal** | **Citations** | **Links** |
| --- | --- | --- | --- | --- |
| Khoury et al., 2007 | The continuum of translation research in genomic medicine: how can we accelerate the appropriate integration of human genome discoveries into health care and disease prevention? | Genetics In Medicine | 511 | 118 |
| Rubio et al., 2010 | Defining Translational Research: Implications for Training | Academic Medicine | 305 | 101 |
| Harris et al., 2009 | Research electronic data capture (REDCap)-A metadata-driven methodology and workflow process for providing translational research informatics support | Journal Of Biomedical Informatics | 23253 | 92 |
| Collins, 2011 | Reengineering Translational Science: The Time Is Right | Science Translational Medicine | 309 | 60 |
| Trochim et al., 2011 | Evaluating Translational Research: A Process Marker Model | Cts-Clinical And Translational Science | 113 | 60 |
| Drolet et al., 2011 | Translational research: understanding the continuum from bench to bedside | Translational Research | 145 | 58 |
| Morris et al., 2011 | The answer is 17 years, what is the question: understanding time lags in translational research | Journal Of The Royal Society Of Medicine | 1267 | 55 |
| van der Lann et al.,2015 | Beyond Bench and Bedside: Disentangling the Concept of Translational Research | Health Care Analysis | 48 | 52 |
| Pober et al., 2001 | Obstacles facing translational research in academic medical centers | Faseb Journal | 95 | 49 |
| Seok et al., 2013 | Genomic responses in mouse models poorly mimic human inflammatory diseases | Proceedings Of The National Academy Of Sciences Of The United States Of America | 2040 | 44 |
| Zerhouni, 2007 | Translational research: Moving discovery to practice | Clinical Pharmacology & Therapeutics | 122 | 44 |
| Littman et al., 2007 | What's next in translational medicine? | Clinical Science | 91 | 37 |
| Fudge et al., 2016 | Optimising Translational Research Opportunities: A Systematic Review and Narrative Synthesis of Basic and Clinician Scientists' Perspectives of Factors Which Enable or Hinder Translational Research | Plos One | 38 | 32 |
| Bazan, 2019 | From lab bench to store shelves: A translational research & development framework for linking university science and engineering research to commercial outcomes | Journal Of Engineering And Technology Management | 8 | 31 |
| Glasgow et al., 2012 | National Institutes of Health Approaches to Dissemination and Implementation Science: Current and Future Directions | American Journal Of Public Health | 476 | 30 |
| Neuwelt et al., 2008 | Strategies to advance translational research into brain barriers | Lancet Neurology | 343 | 30 |
| Neuwelt et al., 2011 | Engaging neuroscience to advance translational research in brain barrier biology | Nature Reviews Neuroscience | 388 | 29 |
| Reis et al., 2010 | Reengineering the National Clinical and Translational Research Enterprise: The Strategic Plan of the National Clinical and Translational Science Awards Consortium | Academic Medicine | 53 | 28 |
| Lander & Atkinson-Grosjean, 2011 | Translational science and the hidden research system in universities and academic hospitals: A case study | Social Science & Medicine | 55 | 26 |
| Whittemore, 2011 | A systematic review of the translational research on the Diabetes Prevention Program | Translational Behavioral Medicine | 106 | 25 |
| Califf & Lars, 2010 | Linking Scientific Discovery and Better Health for the Nation: The First Three Years of the NIH's Clinical and Translational Science Awards | Academic Medicine | 43 | 24 |
| Heller et al., 2009 | Clinical and Translational Science Awards: Can They Increase the Efficiency and Speed of Clinical and Translational Research? | Academic Medicine | 38 | 24 |
| Canuel et al., 2015 | Translational research platforms integrating clinical and omics data: a review of publicly available solutions | Briefings In Bioinformatics | 63 | 23 |
| Chen et al., 2012 | Prospects for translational regenerative medicine | Biotechnology Advances | 56 | 23 |
| Pfund et al., 2014 | Training Mentors of Clinical and Translational Research Scholars: A Randomized Controlled Trial | Academic Medicine | 124 | 23 |
| Brisson et al., 2012 | Translational Research in Pediatrics: Tissue Sampling and Biobanking | Pediatrics | 48 | 21 |
| Albani & Prakken, 2009 | The advancement of translational medicine-from regional challenges to global solutions | Nature Medicine | 32 | 21 |
| Kim et al., 2020 | A bibliometric measure of translational science | Scientometrics | 7 | 21 |
| Kane et al., 2013 | Evaluating Translational Research | Translational Medicine - What, Why And How: An International Perspective | 2 | 20 |
| Maienschein et al., 2008 | The ethos and ethics of translational research | American Journal Of Bioethics | 75 | 20 |
| Surkis et al., 2016 | Classifying publications from the clinical and translational science award program along the translational research spectrum: a machine learning approach | Journal Of Translational Medicine | 26 | 20 |
| Curry, 2008 | Translational science: past, present, and future | Biotechniques | 17 | 19 |
| Keramaris et al., 2008 | Translational research: From benchside to bedside | Injury-International Journal Of The Care Of The Injured | 46 | 19 |
| Wainwright et al., 2006 | From bench to bedside? Biomedical scientists' expectations of stem cell science as a future therapy for diabetes | Social Science & Medicine | 79 | 19 |
| Mullane et al., 2014 | Translational paradigms in pharmacology and drug discovery | Biochemical Pharmacology | 25 | 19 |
| Meyers et al., 2012 | Strengthening the Career Development of Clinical Translational Scientist Trainees: A Consensus Statement of the Clinical Translational Science Award (CTSA) Research Education and Career Development Committees | Cts-Clinical And Translational Science | 45 | 19 |
| Rajan et al., 2012 | Critical Appraisal of Translational Research Models for Suitability in Performance Assessment of Cancer Centers | Oncologist | 21 | 18 |
| Silet et al., 2010 | A National Survey of Mentoring Programs for KL2 Scholars | Cts-Clinical And Translational Science | 43 | 18 |
| Mace et al., 2010 | DIFFERENTIAL REINFORCEMENT OF ALTERNATIVE BEHAVIOR INCREASES RESISTANCE TO EXTINCTION: CLINICAL DEMONSTRATION, ANIMAL MODELING, AND CLINICAL TEST OF ONE SOLUTION | Journal Of The Experimental Analysis Of Behavior | 106 | 18 |
| Podlesnik et al., 2017 | RENEWED BEHAVIOR PRODUCED BY CONTEXT CHANGE AND ITS IMPLICATIONS FOR TREATMENT MAINTENANCE: A REVIEW | Journal Of Applied Behavior Analysis | 74 | 18 |
| Weber, 2013 | Identifying translational science within the triangle of biomedicine | Journal Of Translational Medicine | 35 | 18 |
| Johnson et al., 2010 | An Innovative Program to Train Health Sciences Researchers to Be Effective Clinical and Translational Research Mentors | Academic Medicine | 53 | 17 |
| Grzywacz & Allen, 2017 | Adapting the Ideas of Translational Science for Translational Family Science | Family Relations | 9 | 16 |
| Morgan et al., 2011 | Implementing 'translational' biomedical research: Convergence and divergence among clinical and basic scientists | Social Science & Medicine | 29 | 16 |
| Wehling, 2009 | OPINION Assessing the translatability of drug projects: what needs to be scored to predict success? | Nature Reviews Drug Discovery | 87 | 16 |
| Kyonka & Subramaniam, 2018 | Translating Behavior Analysis: a Spectrum Rather than a Road Map | Perspectives On Behavior Science | 5 | 16 |
| Goldblatt & Lee, 2010 | From bench to bedside: the growing use of translational research in cancer medicine | American Journal Of Translational Research | 74 | 16 |
| Riegman et al., 2008 | Biobanking for better healthcare | Molecular Oncology | 185 | 16 |
| Yu, 2011 | Translational research: current status, challenges and future strategies | American Journal Of Translational Research | 18 | 16 |
| Zhang et al., 2013 | The Quantitative Evaluation of the Clinical and Translational Science Awards (CTSA) Program Based on Science Mapping and Scientometric Analysis | Cts-Clinical And Translational Science | 5 | 16 |
| Llewellyn et al., 2018 | Charting the Publication and Citation Impact of the NIH Clinical and Translational Science Awards (CTSA) Program From 2006 Through 2016 | Academic Medicine | 18 | 16 |
| Rey-Rocha & Martin-Sempere, 2012 | Generating favourable contexts for translational research through the incorporation of basic researchers into hospitals: The FIS/Miguel Servet Research Contract Programme | Science And Public Policy | 10 | 16 |

*More than 50 publications are shared because there were multiple publications with 16 links that tied for the 50^th^ (and previous) spots.

Figure A2. Total citations of the 50* most interlinked publications from Figure 2B. Implementation science directed citation network

| **Publication** | **Title** | **Journal** | **Citations** | **Links** |
| --- | --- | --- | --- | --- |
| Damschroder et al., 2009 | Fostering implementation of health services research findings into practice: a consolidated framework for advancing implementation science | Implementation Science | 5799 | 1277 |
| Graham et al., 2006 | Lost in knowledge translation: Time for a map? | Journal Of Continuing Education In The Health Professions | 2444 | 969 |
| Grimshaw et al., 2012 | Knowledge translation of research findings | Implementation Science | 1250 | 400 |
| Proctor et al., 2011 | Outcomes for Implementation Research: Conceptual Distinctions, Measurement Challenges, and Research Agenda | Administration And Policy In Mental Health And Mental Health Services Research | 2619 | 561 |
| Powell et al., 2015 | A refined compilation of implementation strategies: results from the Expert Recommendations for Implementing Change (ERIC) project | Implementation Science | 1401 | 422 |
| Nilsen, 2015 | Making sense of implementation theories, models and frameworks | Implementation Science | 1548 | 491 |
| Curran et al., 2012 | Effectiveness-implementation Hybrid Designs Combining Elements of Clinical Effectiveness and Implementation Research to Enhance Public Health Impact | Medical Care | 1606 | 405 |
| Morris et al., 2011 | The answer is 17 years, what is the question: understanding time lags in translational research | Journal Of The Royal Society Of Medicine | 1268 | 187 |
| Proctor et al., 2009 | Implementation Research in Mental Health Services: an Emerging Science with Conceptual, Methodological, and Training challenges | Administration And Policy In Mental Health And Mental Health Services Research | 943 | 229 |
| Tabak et al., 2012 | Bridging Research and Practice Models for Dissemination and Implementation Research | American Journal Of Preventive Medicine | 712 | 279 |
| Cane et al., 2012 | Validation of the theoretical domains framework for use in behaviour change and implementation research | Implementation Science | 1922 | 243 |
| Straus et al., 2009 | Defining knowledge translation | Canadian Medical Association Journal | 322 | 198 |
| Waltz et al., 2015 | Use of concept mapping to characterize relationships among implementation strategies and assess their feasibility and importance: results from the Expert Recommendations for Implementing Change (ERIC) study | Implementation Science | 276 | 100 |
| Peters et al., 2013 | Implementation research: what it is and how to do it | Bmj-British Medical Journal | 200 | 137 |
| Palinkas et al., 2011 | Mixed Method Designs in Implementation Research | Administration And Policy In Mental Health And Mental Health Services Research | 506 | 112 |
| Glasgow et al., 2012 | National Institutes of Health Approaches to Dissemination and Implementation Science: Current and Future Directions | American Journal Of Public Health | 476 | 130 |
| Powell et al., 2012 | A Compilation of Strategies for Implementing Clinical Innovations in Health and Mental Health | Medical Care Research And Review | 494 | 148 |
| Meyers et al., 2012 | The Quality Implementation Framework: A Synthesis of Critical Steps in the Implementation Process | American Journal Of Community Psychology | 483 | 111 |
| Rabin et al., 2008 | A glossary for dissemination and implementation research in health | Journal Of Public Health Management And Practice | 332 | 109 |
| Bergstrom et al., 2020 | The use of the PARIHS framework in implementation research and practice-a citation analysis of the literature | Implementation Science | 30 | 107 |
| Eccles et al., 2005 | Changing the behavior of healthcare professionals: the use of theory in promoting the uptake of research findings | Journal Of Clinical Epidemiology | 544 | 111 |
| Tetroe et al., 2008 | Health research funding agencies' support and promotion of knowledge translation: An international study | Milbank Quarterly | 189 | 105 |
| Gagliardi et al., 2016 | Integrated knowledge translation (IKT) in health care: a scoping review | Implementation Science | 180 | 129 |
| Davis et al., 2003 | The case for knowledge translation: shortening the journey from evidence to effect | Bmj-British Medical Journal | 431 | 162 |
| Estabrooks et al., 2006 | A guide to knowledge translation theory | Journal Of Continuing Education In The Health Professions | 241 | 118 |
| Straus et al., 2013 | Knowledge Translation in Health Care: Moving from Evidence to Practice, 2nd Edition | Knowledge Translation In Health Care: Moving From Evidence To Practice, 2Nd Edition | 298 | 118 |
| Greenhalgh & Wieringa, 2011 | Is it time to drop the 'knowledge translation' metaphor? A critical literature review | Journal Of The Royal Society Of Medicine | 304 | 128 |
| Peters et al., 2013 | Implementation research: what it is and how to do it | Bmj-British Medical Journal | 200 | 137 |
| Meyers et al., 2012 | The Quality Implementation Framework: A Synthesis of Critical Steps in the Implementation Process | American Journal Of Community Psychology | 483 | 111 |
| Proctor et al., 2013 | Implementation strategies: recommendations for specifying and reporting | Implementation Science | 672 | 235 |
| Waltz et al., 2015 | Use of concept mapping to characterize relationships among implementation strategies and assess their feasibility and importance: results from the Expert Recommendations for Implementing Change (ERIC) study | Implementation Science | 276 | 100 |
| Kirk et al., 2016 | A systematic review of the use of the Consolidated Framework for Implementation Research | Implementation Science | 278 | 121 |
| Damschroder et al., 2013 | Evaluation of a large-scale weight management program using the consolidated framework for implementation research (CFIR) | Implementation Science | 255 | 103 |
| Scott et al., 2012 | Systematic review of knowledge translation strategies in the allied health professions | Implementation Science | 201 | 85 |
| Straus et al., 2011 | Knowledge translation is the use of knowledge in health care decision making | Journal Of Clinical Epidemiology | 211 | 97 |
| Dobbins et al., 2009 | A description of a knowledge broker role implemented as part of a randomized controlled trial evaluating three knowledge translation strategies | Implementation Science | 260 | 96 |
| Waltz et al., 2019 | Choosing implementation strategies to address contextual barriers: diversity in recommendations and future directions | Implementation Science | 184 | 92 |
| LaRocca et al., 2012 | The effectiveness of knowledge translation strategies used in public health: a systematic review | Bmc Public Health | 176 | 92 |
| Proctor et al., 2013 | The implementation research institute: training mental health implementation researchers in the United States | Implementation Science | 199 | 90 |
| Kothari & Wathen, 2013 | A critical second look at integrated knowledge translation | Health Policy | 139 | 87 |
| Grimshaw et al., 2006 | Toward evidence-based quality improvement - Evidence (and its limitations) of the effectiveness of guideline dissemination and implementation strategies 1966-1998 | Journal Of General Internal Medicine | 472 | 82 |
| Weiner et al., 2017 | Psychometric assessment of three newly developed implementation outcome measures | Implementation Science | 428 | 81 |
| Nilsen & Bernhardsson, 2019 | Context matters in implementation science: a scoping review of determinant frameworks that describe contextual determinants for implementation outcomes | Bmc Health Services Research | 196 | 80 |
| Stetler et al., 2006 | The role of formative evaluation in implementation research and the QUERI experience | Journal Of General Internal Medicine | 328 | 77 |
| Dobbins et al., 2009 | A randomized controlled trial evaluating the impact of knowledge translation and exchange strategies | Implementation Science | 153 | 77 |
| Davis & D'Lima, 2020 | Building capacity in dissemination and implementation science: a systematic review of the academic literature on teaching and training initiatives | Implementation Science | 21 | 72 |
| Lavis, 2006 | Research, public policymaking, and knowledge-translation processes: Canadian efforts to build bridges | Journal Of Continuing Education In The Health Professions | 187 | 72 |
| Bornbaum et al., 2015 | Exploring the function and effectiveness of knowledge brokers as facilitators of knowledge translation in health-related settings: a systematic review and thematic analysis | Implementation Science | 132 | 72 |
| Lewis et al., 2018 | From classification to causality: Advancing Understanding of Mechanisms of change in implementation science | Frontiers In Public Health | 171 | 71 |
| Lang et al., 2007 | Knowledge translation: Closing the evidence-to-practice gap | Annals Of Emergency Medicine | 191 | 69 |
| Helfrich et al., 2010 | A critical synthesis of literature on the promoting action on research implementation in health services (PARIHS) framework | Implementation Science | 185 | 69 |
| Keith et al., 2017 | Using the Consolidated Framework for Implementation Research (CFIR) to produce actionable findings: a rapid-cycle evaluation approach to improving implementation | Implementation Science | 239 | 69 |
| Bowen & Graham, 2013 | From Knowledge Translation to Engaged Scholarship: Promoting Research Relevance and Utilization | Archives Of Physical Medicine And Rehabilitation | 111 | 69 |
| Jull et al., 2017 | Community-based participatory research and integrated knowledge translation: advancing the co-creation of knowledge | Implementation Science | 191 | 69 |

*More than 50 publications are shared because there were multiple publications with 69 links that tied for 50^th^ spot.
